# Two DsbA Proteins Are Important for *Vibrio parahaemolyticus* Pathogenesis

**DOI:** 10.3389/fmicb.2019.01103

**Published:** 2019-05-16

**Authors:** Chun-qin Wu, Ting Zhang, Wenwen Zhang, Mengting Shi, Fei Tu, Ai Yu, Manman Li, Menghua Yang

**Affiliations:** ^1^Key Laboratory of Applied Technology on Green-Eco-Healthy Animal Husbandry of Zhejiang Province, College of Animal Science and Technology, Zhejiang A&F University, Hangzhou, China; ^2^Department of Animal Science, Wenzhou Vocational College of Science and Technology, Wenzhou, China; ^3^Faculty of Health Sciences, University of Macau, Macau, China; ^4^College of Life Science, Nanjing Agricultural University, Nanjing, China

**Keywords:** *Vibrio parahaemolyticus*, DsbA, reductase, virulence, pathogenesis

## Abstract

Bacterial pathogens maintain disulfide bonds for protein stability and functions that are required for pathogenesis. *Vibrio parahaemolyticus* is a Gram-negative pathogen that causes food-borne gastroenteritis and is also an important opportunistic pathogen of aquatic animals. Two genes encoding the disulfide bond formation protein A, DsbA, are predicted to be encoded in the *V. parahaemolyticus* genome. DsbA plays an important role in *Vibrio cholerae* virulence but its role in *V. parahaemolyticus* is largely unknown. In this study, the activities and functions of the two *V. parahaemolyticus* DsbA proteins were characterized. The DsbAs affected virulence factor expression at the post-translational level. The protein levels of adhesion factor VpadF (VP1767) and the thermostable direct hemolysin (TDH) were significantly reduced in the *dsbA* deletion mutants. *V. parahaemolyticus* lacking *dsbA* also showed reduced attachment to Caco-2 cells, decreased β-hemolytic activity, and less toxicity to both zebrafish and HeLa cells. Our findings demonstrate that DsbAs contribute to *V. parahaemolyticus* pathogenesis.

## Introduction

*Vibrio parahaemolyticus* is a Gram-negative halophilic bacterium that lives in estuarine, marine and coastal surroundings. It is one of the leading causes of human food borne gastroenteritis due to consumption of raw or under-cooked seafood (Yeung and Boor, 2004; Su and Liu, 2007; Zhang and Orth, 2013), and is also one of the major threats in aquaculture (Tey et al., 2015). *V. parahaemolyticus* can cause acute hepatopancreas necrosis disease (AHPND) in shrimp and is one of the major pathogens of cultured mud crabs (Xiao et al., 2017; Zhang et al., 2019).

*Vibrio parahaemolyticus* secretes virulence factors to establish successful infection in the host (Broberg et al., 2011; Letchumanan et al., 2014). Virulence factors that contain free thiol-groups (cysteine residues) require disulfide bonds for proper folding and function. Studies of oxidoreductases are therefore important for understanding bacterial pathogenesis and for developing novel therapeutics (Heras et al., 2009). Disulfide bond formation pathways in Gram-negative bacteria have been well studied in *Escherichia coli* (Kadokura and Beckwith, 2009; Depuydt et al., 2011). Disulfide bond formation protein A, DsbA, introduces disulfide bonds into proteins between consecutive cysteine residues as they pass through the inner membrane translocation system (Bardwell et al., 1991; Berkmen et al., 2005; Kadokura and Beckwith, 2009).

The Dsb redox system plays a pivotal role in the virulence of many pathogens (Braun et al., 2001; Heras et al., 2009; Qin et al., 2009; Ren et al., 2014; Mariano et al., 2018). *Francisella tularensis* requires the DsbA redox system to promote the proper folding of virulence proteins (Qin et al., 2009; Ren et al., 2014). *Pseudomonas aeruginosa* DsbA is required for the expression of elastase, exotoxin A, protease IV, and is also required for the formation of a functional type III secretion system (Braun et al., 2001; Ha et al., 2003). *Serratia marcescens* DsbA is required for virulence and for proper deployment of the T6SS (Mariano et al., 2018). We previously found that DsbA is indispensable to *Vibrio cholerae* pathogenesis. A *dsbA* deletion mutant of *V. cholerae* did not colonize the intestine because the expression of the master virulence gene regulator ToxT was abolished in the *dsbA* deletion mutant (Yang et al., 2013; Xue et al., 2016).

Here, we investigated the role of *V. parahaemolyticus* DsbA in pathogenesis. Two DsbA genes are predicted to be encoded in the *V. parahaemolyticus* genome. We analyzed the reductase activities and the redox potential of the purified DsbA proteins and characterized the virulence properties of single and double *dsbA* mutants. Deleting the dsbA genes affected *V. parahaemolyticus* virulence factor expression at the post-translational level.

## Materials and Methods

### Bacterial Strains, Plasmids, and Media

The bacterial strains, plasmids, and oligonucleotides used in this study are summarized in Tables 1, 2. All *V. parahaemolyticus* strains used in this study were derived from the HZ strain, a clinical isolate from the Zhejiang Provincial Center for Disease Control and Prevention, Zhejiang, China (Yu et al., 2015). *Escherichia coli* strains DH5α, BL21, and CC118λpir were used for general manipulation of plasmids, prokaryotic expression of proteins, and mobilization of plasmids into *V. parahaemolyticus*, respectively. The bacterial strains were grown at 37°C in Luria-Bertani (LB) broth with 1% NaCl (Sambrook et al., 1989) (for *E. coli*) or LB-NaCl (for *V. parahaemolyticus*) which is LB broth supplemented with 3% NaCl containing appropriate antibiotics. Plasmids for overexpressing VpDsbAs or VpDsbB or other proteins in *V. parahaemolyticus* were constructed by cloning the PCR-amplified coding regions into pBAD24Cm (Guzman et al., 1995) and introduced into *V. parahaemolyticus* by conjugation with the helper strain of CC118λpir (Yu et al., 2015). Plasmids for overexpressing VpDsbAs in *E. coli* were constructed by cloning the coding regions into pacyc177 which has been modified to obtain an arabinose operon (Chang and Cohen, 1978) and introduced into *E. coli* strain by electroporation (Sambrook et al., 1989).

**Table 1:** Bacterial strains and primers used in this study.

Strain	Relevant characteristics	References
*V. parahaemolyticus* strains		
HZ	Wild type (WT), clinical strain	Yu et al., 2015
YA1	Δ*VpdsbA1, VpdsbA1* (*vp3054*) deletion	This study
YA2	Δ*VpdsbA2, VpdsbA2* (*vpa1271*) deletion	This study
YA3	Δ*VpdsbA1/2, VpdsbA1* and *VpdsbA2* deletion	This study
YA4	Δ*tdhAS, tdhA* (*vpa1314*) and *tdhS* (*vpa1378*) deletion	This study
YA5	Δ*tdhAS/vp1696, tdhA*, *tdhS* and *VP1696* deletion	This study
YA6	Δ*tdhAS/vtrA, tdhA*, *tdhS* and *vtrA* (*vpa1332*) deletion	This study
ZT1	Δ*tdhAS/vtrA/VpdsbA1/2, tdhA*, *tdhS*, *vtrA, VpdsbA1* and *VpdsbA2* deletion	This study
ZT2	Δ*tdhAS/VP1696/VpdsbA1/2, tdhA*, *tdhS*, *VP1696, VpdsbA1* and *VpdsbA2* deletion	This study
ZT3	Δ*VpdsbA1, VpdsbA1* deletion containing P*_BAD_*-VpdsbA1	This study
ZT4	Δ*VpdsbA2, VpdsbA2* deletion containing P*_BAD_*-VpdsbA2	This study
ZT5	Δ*VpdsbA1, VpdsbA1* deletion containing P*_BAD_*-VpadF-cFLAG	This study
ZT6	Δ*VpdsbA2, VpdsbA2* deletion containing P*_BAD_*-VpadF-cFLAG	This study
ZT7	Δ*VpdsbA1/2, VpdsbA1* and *VpdsbA2* deletion containing P*_BAD_*-VpadF-cFLAG	This study
ZT8	Δ*VpdsbA1, VpdsbA1* deletion containing P*_BAD_*-MAM7-cFLAG	This study
ZT9	Δ*VpdsbA2, VpdsbA2* deletion containing P*_BAD_*-MAM7-cFLAG	This study
ZT10	Δ*VpdsbA1/2, VpdsbA1* and *VpdsbA2* deletion containing P*_BAD_*-MAM7-cFLAG	This study
*Escherichia coli* strains		
DH5α	*E. coli* for cloning strain	Hanahan, 1983
BL21	*E. coli* for protein expression and purification strain	Karimova et al., 1998
CC118λpir	*E. coli* for mobilization of plasmids into *V. parahaemolyticus*	Yu et al., 2015

**Table 2:** Primer sequences used for cloning in this study.

Cloning	Primer sequence (5′→3′)
P*_BAD_-VpdsbA1*	F: CCGGAATTCATGAAAAAACTGTTCGCACTGTT
	R: CATTGCTGCAGTTATTTTAGCGTTAGCAAGTAGTTCAC
P*_BAD_-VpdsbA2*	F: GCTCTAGAGGGCTAGCAGGAGGAATTCATG
	R: ACATGCATGCTTATAGCGTTAGCAGATAGTTCACCAATTC
*VpdsbA1* dele	P1: CGCGGATCCCAATTTACCGATGAAGAGCGC
	P2: CAGGAACTACTTCAAGATCCAATACTTTGTAATGTT
	P3: GATCTTGAAGTAGTTCCTGCGGTTATCGTAAATAAC
	P4: CGAGCTCTTAACGCTTCTTTTGTGATCCTC
*VpdsbA2* dele	P1: CGCGGATCCAAACACATCGGTCACCCAAAG
	P2: GGACGTTGAGAAAGGCGTTCCTGGCGTC
	P3: AGGAACGCCTTTCTCAACGTCCAACACTTTG
	P4: CGAGCTCCATTTGGAACGTAAGCATCTGC
*vp1314* dele	P1: GAATTCCTGCAGCCCGGGGGATCCCTGAATTAGTAGAG TTAATC
	P2: CATTTTACTTGGTCGAACAACAAACAATATCTCATCAG
	P3: TGTTGTTCGACCAAGTAAAATGTATTTGGATGAAAC
	P4: CTAAAGGGAACAAAAGCTGGAGCTCCTACCGCTAAATGC
*vp1378* dele	P1: GAATTCCTGCAGCCCGGGGGATCCGTGGAAACAAGGCAAGC
	P2: CATTTTACTTGGTCGAACAACAAACAATATCTCATCAG
	P3: TGTTGTTCGACCAAGTAAAATGTATTTGGATGAAAC
	P4: CTAAAGGGAACAAAAGCTGGAGCTCGTCTGATATCCGT GAAC
*vp1696* dele	P1: GAATTCCTGCAGCCCGGGGGATCCGTGTGGTTTCGA TGTCGTC
	P2: GTTTACAAAGCGCGCCGAACAGTTCGCATGTTC
	P3: CTGTTCGGCGCGCTTTGTAAACGTGCAGTACTG
	P4: CTAAAGGGAACAAAAGCTGGAGCTCGCTGATCCTTTGT TGCTAC
*vpa1332* dele	P1: CGCGGATCCCTATTATGTTTAAATCCACCATCTCCTG
	P2: TTGTTTTATCCAAGGCGAGGAGCACGAGATG
	P3: CCTCGCCTTGGATAAAACAATATCTTTTAGC
	P4: CGCGGATCCGGCTTGCTGCAGACGGTATTTAG
T7-VpDsbA1	P1: CATGCCATGGCGCAATTCAAAGAAGG
	P2: CCCAAGCTTTTTTAGCGTTAGCAAGTAGTTCACC
T7-VpDsbA2	P1: GAATTCCATATGGCTCAATTTGAAGAAGGTAAAC ACTAC
	P2: CCCAAGCTTTTAGTGATGATGATGATGATGTAGCGTTAGCAG
T7-VPA1314	P1: CATGCCATGGGCTTTGAGCTTCCATCTGTCCCTTTTC
	P2: CCCAAGCTTTTGTTGATGTTTACATTCAAAAAACG

### Construction of the *dsbA* and *dsbB* Mutants

In-frame deletion strains used in this study were described in previous publications (Yu et al., 2015; Jiang et al., 2018). In-frame deletions of *VpdsbA1* (*vp3054*), *VpdsbA2* (*vpa1271*)*, VpdsbB* (*vp2073*) or *EcDsbA* were constructed by cloning the regions flanking the target genes into suicide vector pDS132 (Philippe et al., 2004) containing a *sacB* counter selectable marker (Metcalf et al., 1996). The resulting plasmids were introduced into *V. parahaemolyticus* or *E. coli* by conjugation (Boyer, 1966; Yu et al., 2015) and deletion mutants were selected for double homologous recombination events (Kaniga et al., 1991).

### Zebrafish Virulence Evaluation

Zebrafish virulence assays were designed based on a previous publication (Paranjpye et al., 2013). All animal experiments were carried out in strict accordance with the animal protocols that were approved by the Institutional Animal Care and Use Committee of Zhejiang A&F University (Permit Number: ZJAFU/IACUC_2011-10-25-02). Care and feeding of zebrafish followed established protocols^1^. Zebrafish were obtained from a commercial supplier between 5 and 6 months old and were raised at our animal facility at Zhejiang A&F University for at least 2 weeks before challenge experiments following previously published protocols (Paranjpye et al., 2013). For challenge experiments, zebrafish were injected intraperitoneally using a repeater dispenser (Hamilton #83700) and a 33 gauge needle (Hamilton 1750LTSN, 33/0.3759/PT4) following previously published protocols (Lefebvre et al., 2009). Ten zebrafish per group were inoculated with each strain at a concentration of 10^7^ cfu 10 μl^−1^ of the inoculum and observed every 4 or 8 h for 48 h. Control fish injected with 10 μl PBS were included with each experiment. Experiments were repeated at least once. Tris-buffered tricaine at a concentration of 320 μg ml^−1^ was used to kill the fish on completion of the experiment. All aquaria and water were disinfected with 20% sodium hypochlorite after each experiment. Filters from air pumps were sterilized by autoclaving.

### DsbA Cloning, Expression, and Purification

*Vibrio parahaemolyticus VpdsbA1*, *VpdsbA2* or *VPA1314* genes without the signal peptide sequence were amplified from the genomic DNA which was purified by QIAamp DNA Mini Kit (Qiagen) from bacterial culture. The PCR products without the signal peptide sequence were inserted into a modified pET-28a (Novagen, Inc.) vector encoding an N-terminal His_6_ tag. *E. coli* BL21(DE3)/pLysS (Karimova et al., 1998) cells were transformed with the plasmid containing the target gene and transformed cells were used for protein expression by autoinduction (Thompson et al., 1994). Proteins were expressed and purified on nickel columns according to the manufacturer’s instructions (Invitrogen).

### Antibody Preparation

Ten milligrams of each protein, VpDsbA1, VpDsbA2, and VPA1314 (TDH) purified as described above were used as antigens, and then sent to GenScript Inc. for polyclonal antibodies preparation by immunizing rabbits. Antibody effectiveness was detected by Western blotting of each specific purified protein a week after the fourth immunization.

### Insulin Reduction Assay

The protein disulfide reductase activity of VpDsbA was measured *in vitro* using the insulin-reduction assay in the presence of dithiothreitol (DTT) (Holmgren, 1979). Each DsbA protein (10 μM) was mixed with buffer consisting of 100 mM sodium phosphate, 2 mM ethylenediaminetetraacetic acid (EDTA), 0.33 mM DTT. Insulin (170 μM) was added immediately before measurements were made. Insulin reduction by DTT was monitored spectrophotometrically at 650 nm.

### Redox Potential Determination

VpDsbA redox potential assays were performed essentially as described by Walden et al. (2012). VpDsbA (2 μM) was incubated in fully degassed buffer consisting of 100 mM sodium phosphate, 1 mM EDTA pH 7.0 containing 1 mM oxidized glutathione (GSSG; Sigma–Aldrich, United States) and a range of reduced glutathione (GSH) concentrations (0.1–2.0 mM) for 24 h at room temperature. After incubation, the reactions were stopped with 10% trichloroacetic acid (TCA) and the precipitated protein pellets were collected by centrifugation at 16,000 × *g* for 30 min at 4°C. The pellets were washed with 100% ice-cold acetone and dissolved in a buffer consisting of 50 mM Tris-HCl pH 7.0, 1% sodium dodecyl sulfate (SDS), 10 mM 4-acetamide-4′-maleimidylstilbene-2,2′-disulfonate (AMS) to label the free thiols. Separation of the reduced and oxidized forms was performed on 12% SDS-polyacrylamide gels under denaturing conditions. Gels were stained with Coomassie Brilliant Blue and scanned. The relative intensity of the reduced and oxidized forms was analyzed using ImageJ^2^. The fraction of the reduced protein was plotted against the ratio [GSH]^2^/[GSSG]. The equilibrium constant *K*_eq_ was calculated using the equation:

$$R=\frac{{\left[\mathrm{GSSG}\right]}^{2}}{\left[\mathrm{GSH}\right]}/(K_{\mathrm{eq}}+\frac{{\left[\mathrm{GSSG}\right]}^{2}}{\left[\mathrm{GSH}\right]})$$

where *R* is the fraction of reduced protein at equilibrium. The standard redox potential was calculated using the Nernst equation,

$$E^{0^{\prime }}=E_{\mathrm{GSSG}/\mathrm{GSH}}^{0^{\prime }}-\frac{\mathrm{RT}}{\mathrm{nF}}\ln K_{\mathrm{eq}}$$

where *E*^0′^_*GSH/GSSG*_ is the standard potential of −240 mV (Gilbert, 1995), *R* is the universal gas constant 8.314 J K^−1^mol^−1^, *T* is the absolute temperature in Kelvin, *n* is the number of electrons transferred, *F* is the Faraday constant 9.648 × 10^4^ C mol^−1^ and *K*_eq_ is the equilibrium constant.

### Real-Time Quantitative PCR (RT-qPCR) Analysis

Overnight cultures of *V. parahaemolyticus* WT or *dsbA* mutants were subcultured at a dilution of 1:100 in LB-NaCl medium and incubated without shaking at 37°C for 4 h. Total RNA was purified from bacterial cultures using TRIzol reagent (Invitrogen), DNase digestion and RNA reverse transcription was performed by using the PrimeScript RT reagent Kit with gDNA Eraser (TaKara). Quantitative real-time qPCR was performed in 20 μl reaction mixtures containing SYBR quantitative PCR mix (Toyobo) to measure the transcriptional levels of genes of interest using the Mx3000P PCR detection system (Agilent) with primers specific for tested genes. The 16s rRNA gene were used as internal controls in all reactions.

### Spot Titers Cadmium Resistance

Cadmium resistance was performed to quantify the relative disulfide oxidase activity of the strains *in vivo* as described in Xue et al. (2016). Briefly, strains were grown overnight in LB and diluted 1:100 into fresh LB media with appropriate antibiotics. Strains were grown to mid-logarithmic phase at 37°C and serially diluted into phosphate buffer salt (PBS). A 5 μl aliquot of each dilution was plated onto LB plates with a cadmium gradient. Cells were grown at 37°C overnight. All spot titers were performed at least in triplicate.

### Isothermal Titration Calorimetry

Affinity and thermodynamics of binding between taurocholate (TC) and VpDsbA proteins were assessed by isothermal titration calorimetry (ITC) using a VP-ITC instrument (MicroCal^TM^, GE Healthcare) according to the protocols as described in Xue et al. (2016). Briefly, the sample cell was loaded with 1.5 ml of purified protein at 200 μM concentration in PBS. The syringe was filled with TC in the same buffer as that was used to dilute the proteins at a concentration of 4 mM. Titrations were conducted at 25°C using 25 consecutive injections of 10 μl each delayed by 300 s with a stirring speed of 307 rpm. As a control for background noise, titration of TC into a solution containing the buffer only was performed. The association constant (*K_a_* = 1/*K_d_*), free energy (ΔG), and enthalpy change (ΔH) and entropy change (ΔS) were calculated by fitting the data to a single-site binding model using the MicroCal Origin software (Origin 7.0 SR4 version7.0552β). Parameters reported include the mean ± SD across three replicates. The calculated *c*-value for these measurements is 12.

### Motility Assay in Soft Agar

Motility assay was performed essentially on soft LB (for *E. coli* strains) or LB-NaCl (for *V. parahaemolyticus* strains) agar (0.25%) as described (Paxman et al., 2009). *E. coli* or *V. parahaemolyticus* strains were grown overnight on LB or LB-NaCl agar and a single colony of each strain was inserted into soft agar by tooth pick and incubated at 37°C for 8–12 h. Motility was assessed by examining migration of bacteria through agar from the center toward the periphery of the colony. The diameter of the motility circle was analyzed using ImageJ (see text footnote 2).

### VpDsbA Oxidized by VpDsbB Present in Membranes

VpDsbA oxidized by VpDsbB *in vitro* was performed as previously described (Xue et al., 2016). *V. parahaemolyticus* strain Δ*dsbA* harboring a plasmid encoding VpDsbB under the control of an arabinose inducible promoter was cultured at 37°C with 200 rpm shaking in LB-NaCl medium until OD_600_≈0.5. 0.2% of arabinose was added and bacteria were kept cultured in the same conditions for another 16 h. Membranes were prepared according to Kovach et al. (1995). Purified DsbA was reduced by incubation in 50 mM DTT for 10 min at 4°C. DTT was then removed by gel filtration on PD-10 Sephadex columns (GE). Reduced DsbA was stored at −80°C in the presence of 0.1 mM EDTA, pH 8.0. VpDsbB membrane suspension (1 mg/ml) was mixed with 10 mM ubiquinone 1 (UQ1) with or without different concentrations of TC as indicated in PBS. Reactions were started right after 2 mM of reduced VpDsbA was added. TCA (10%) was added at different time points to stop the reaction and protein was precipitated at 4°C overnight. Precipitated proteins were treated with AMS as described. Negative controls were the membrane of *V. parahaemolyticus* Δ*dsbA/dsbB*.

### Cell Adhesion Assay

Caco-2 cells (Sigma-Aldrich Inc.) were maintained in Dulbecco’s Modified Eagle Medium (DMEM, Invitrogen) supplemented with 10% fetal bovine serum (FBS, Invitrogen) at 37°C in 5% CO_2_. Adhesion assays were carried out as previously described (Liu and Chen, 2015). Briefly, Caco-2 cells were infected with freshly prepared *V. parahaemolyticus* cultures of tested strains at a multiplicity of infection (MOI) of 10. As a control, bacterial cells were added to the empty wells of the cell culture plates and incubated for the same time as the binding experiment to determine the final total number of *V. parahaemolyticus* for the experiment. After 1 h infection, cells were washed three times with PBS and then lysed with 1% Triton X-100 at 37°C for 10 min. The cell lysates and control bacteria were serially diluted and plated on LB-NaCl agar. Attachment rate was calculated by dividing bound bacteria to the total bacterial load.

### Kanagawa Hemolytic (KH) Activity Assay

Kanagawa hemolytic (KH) hemolytic activity was determined as described in Chun et al. (1975). Briefly, Wagatsuma blood agar (WBA) (Chun et al., 1975) contained 10% washed rabbit erythrocytes was prepared freshly before each assay. Bacteria from freshly prepared *V. parahaemolyticus* cultures of test strains were pelleted by centrifugation and washed with PBS. ∼10^8^ CFU in 10 μl bacterial suspension were dropped on WBA plates, and the result was observed after incubation for 24 h at 37°C. Well-defined, clear hemolysis around the bacterial growth was recorded and the hemolysis around area was analyzed using ImageJ (see text footnote 2).

### Cytotoxicity Assay

The cytotoxic assays were performed as described previously (Hiyoshi et al., 2010). HeLa cells (Thermo Fisher Scientific Inc.) were seeded in 96-well plates. The overnight cultured strains were sub-cultured at the ratio 1:50 to the fresh LB-NaCl broth medium and grow at 37°C to OD_600_≈0.5. Bacteria were pelleted by centrifugation and washed with PBS, then suspended in two volumes of unsupplemented DMEM (DMEM without FBS). Before infection, HeLa cells were also washed with unsupplemented DMEM. Infection was performed at an MOI of 10. After infection, the release of lactate dehydrogenase (LDH) into the medium was quantified at each indicated time point with a CytoTox96 kit (Promega, Madison, WI, United States) used according to the manufacturer’s instructions.

### Statistical Analysis

All data are presented as the mean ± SD of three determinations in each experimental condition. Statistical significance was determined using one-way ANOVA. *P* < 0.05 was considered statistically significant.

## Results

### *V. parahaemolyticus* Encodes Two *dsbA* Genes

**FIGURE 1:**
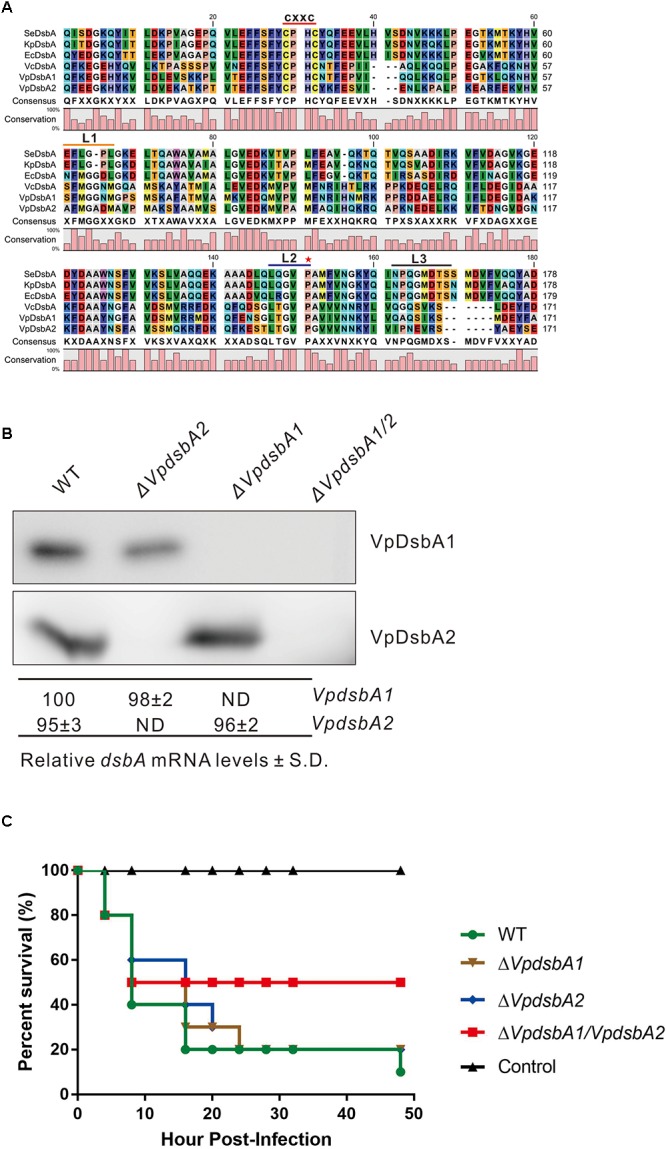
VpDsbAs modulate *V. parahaemolyticus* virulence in zebrafish. **(A)** Amino acid alignment of VpDsbA1, VpDsbA2, VcDsbA, and EcDsbA. The red line indicates the conserved CXXC motif. Three loop sequences of L1, L2, and L3 are indicated as an orange, blue, and black line, respectively, and a red star indicates the predicted *cis*Pro. **(B)** *Top*, analysis of VpDsbA1 and VpDsbA2 expression level. *V. parahaemolyticus* WT, Δ*VpdsbA1*, Δ*VpdsbA2*, and Δ*VpdsbA1/2* were grown in LB-NaCl until OD_600_≈0.8. Cell lysates (1 mg) were separated by SDS-PAGE and VpDsbA1 or VpDsbA2 was detected by the Western blot using antiserum specific for either VpDsbA1 or VpDsbA2. Blot shown is representative of at least three separate experiments. *Bottom*, analysis of *VpdsbA1* and Δ*VpdsbA2* mRNA levels by qRT-PCR. RNA was purified from freshly prepared cultures grown in LB-NaCl. The % *dsbA* mRNA levels ± standard deviation (SD) were normalized to 16S RNA for each strain and relative to WT (set to 100%). ND, none detected. **(C)** Survival curves (Kaplan–Meier) of zebrafish following intraperitoneal challenges of *V. parahaemolyticus* WT, Δ*VpdsbA1*, Δ*VpdsbA2*, and Δ*VpdsbA1/2* strains.

VP3054 (NP_799433.1) and VPA1271 (NP_800781.1) are predicted to be DsbA homologs in *V. parahaemolyticus*. The amino acid sequences of VP3054 and VPA1271 were compared and thioredoxin-fold molecular features were observed (Figure 1A). Both VP3054 and VPA1271 show about 40% amino acid sequence identity to *E. coli* DsbA (EcDsbA), *Klebsiella pneumonia* DsbA (KpDsbA) or *Salmonella enterica* DsbA (SeDsbA), and the identities are even higher when compared to *V. cholerae* DsbA from (VcDsbA). VP3054 shows 79% identity to VcDsbA while VPA1271 is 59% identical (Figure 1A). Both VP3054 and VPA1271 encode a DsbA-like domain, including a conserved CXXC active-site motif (CPHC in this case) and a putative *cis*Pro motif (Ren et al., 2009) (Figure 1A). Comparison of loop sequences on the catalytic face of these DsbA proteins revealed that both the two DsbAs from *V. parahaemolyticus* should belong to Class-Ia DsbA (McMahon et al., 2014; Totsika et al., 2018). To differentiate these two proteins, we named VP3054 as VpDsbA1 and VPA1271 as VpDsbA2.

We found that both *VpdsbA1* and *VpdsbA2* are expressed in *V. parahaemolyticus* (Figure 1B), and mutating one gene did not appreciably affect the expression of the other gene. In a zebrafish infection model, Δ*VpdsbA1/2* had significantly reduced virulence while both Δ*VpdsbA1* and Δ*VpdsbA2* were as virulent as the wild type strain (Figure 1C). Each mutant grew similarly to the wild-type strain in broth culture (Supplementary Figure S1). These results suggest that both VpDsbA1 and VpDsbA2 are functional in *V. parahaemolyticus* and they may function redundantly, because a virulence defect was only seen in the double mutant.

### Redox Properties of VpDsbA1 and VpDsbA2

**FIGURE 2:**
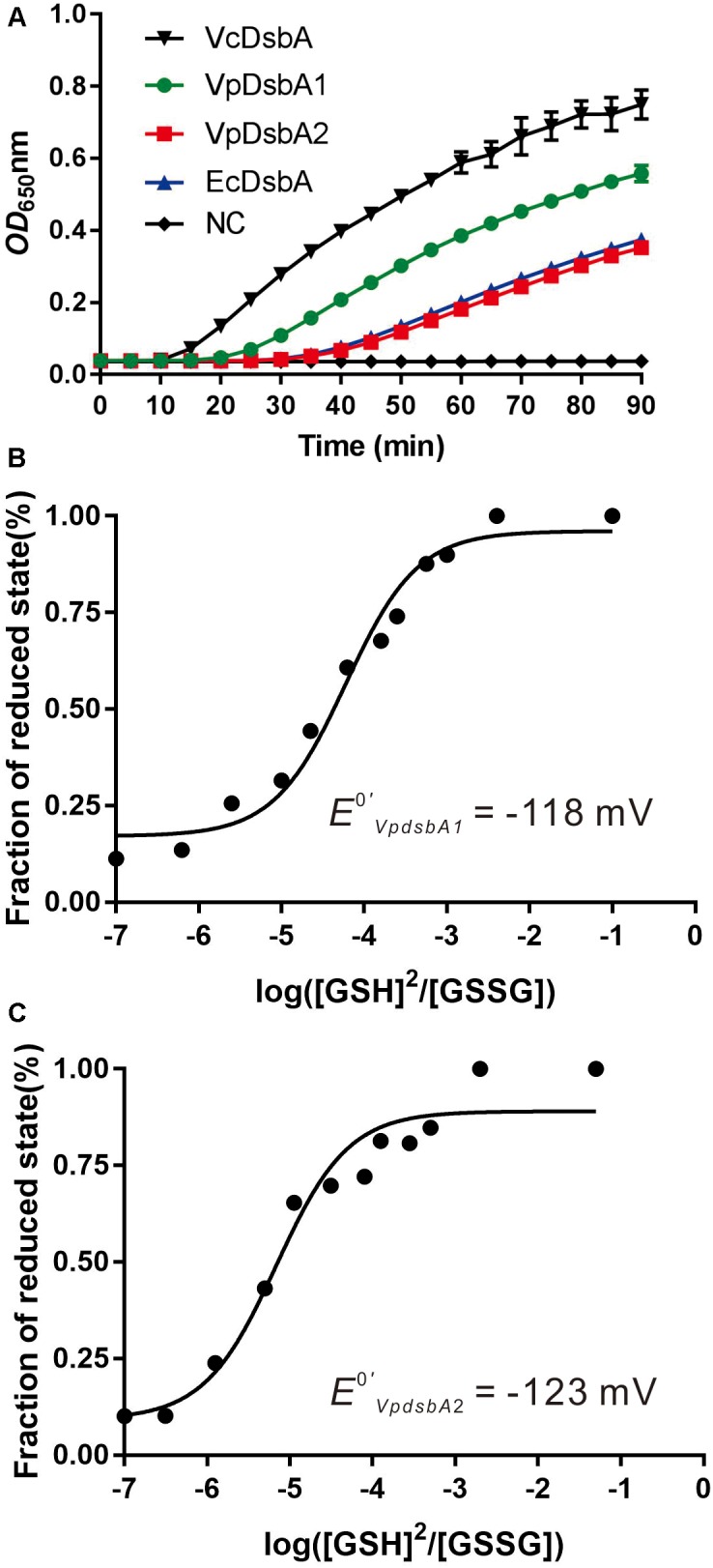
Reductase activity and redox potential of VpDsbAs. **(A)** Insulin (170 μM) and 0.33 mM DTT was incubated with 10 μM of each DsbA protein. The reduction of insulin was measured by monitoring the increase in absorbance at OD_650_. Reaction without reductase protein is used as negative control (NC). **(B,C)** Characterization of the redox potential of VpDsbA1 **(B)** and VpDsbA2 **(C)**. Non-linear fit to the fraction of reduced VpDsbA at different ratios of reduced:oxidized glutathione. This fit was used to obtain the equilibrium constant *K*_eq_ and the redox potential [calculated relative to the GSH/GSSG standard potential of –240 mV (Gilbert, 1995)]. The curve was fit to the averaged data from three replicate experiments.

To investigate the enzyme activity of VpDsbA1 and VpDsbA2, we performed a standard reductase activity assay using folded insulin as the substrate and DTT as the electron donor (Holmgren, 1979). Insulin comprises A and B chains which are linked by two disulfide bonds, and the reduction of the disulfide bonds leads to B-chain precipitation, which can be monitored as an increase in absorbance at 650 nm. B chain precipitation and a consequent increase in the OD_650_ was measurable after 22 min with EcDsbA (Figure 2A). B chain precipitation was observed after 15 min with VpDsbA1 and after 22 min with VpDsbA2. Insulin reduction was observed after 10 min with VcDsbA.

To better characterize the redox properties of VpDsbA1 and VpDsbA2, we determined the standard redox potential of these two proteins relative to the redox potential of GSH/GSSG (−240 mV) (Gilbert, 1995). By calculating the fraction of reduced VpDsbA at different concentrations of [GSH]^2^/[GSSG], the equilibrium constants were determined (Figure 2B,C). The *K*_eq_ for VpDsbA1 was 8.4+0.06 × 10^−5^ M and VpDsbA2 was 1.2+0.03 × 10^−4^ M. The corresponding redox potentials of VpDsbA1 and VpDsbA2 are −118 mV and −123 mV rat pH 7.0. Thus, VpDsbA1 is relatively oxidizing, with a redox-potential value similar to that of the thioredoxin-fold proteins, VcDsbA (*E*^0′^ = −116 mV) (Walden et al., 2012) and the redox-potential value of VpDsbA2 is similar to that of VcDsbA (*E*^0′^ = −122 mV) (Huber-Wunderlich and Glockshuber, 1998).

### VpDsbAs Function as Oxidases in *V. parahaemolyticus*

**FIGURE 3:**
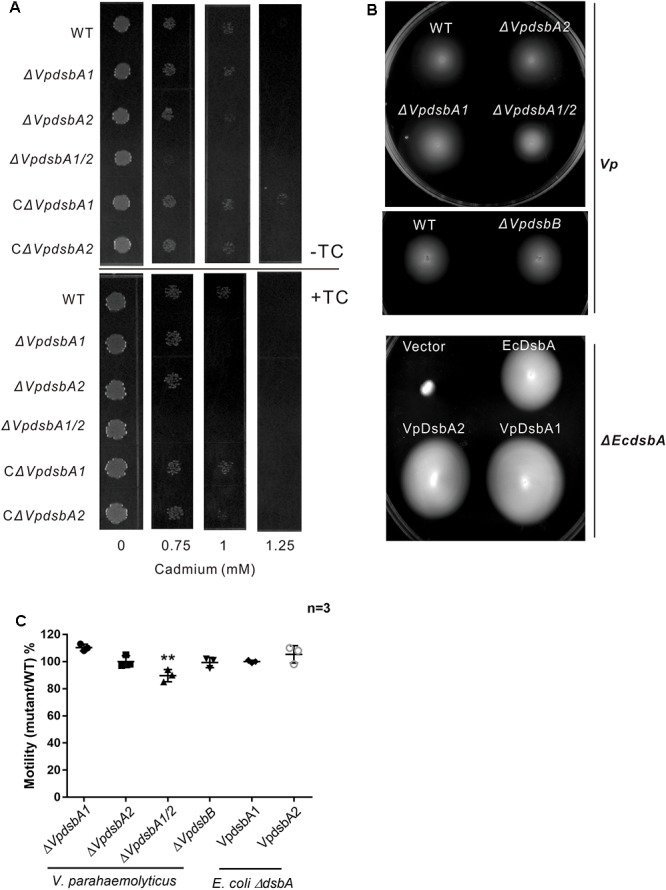
VpDsbA *in vivo* activity assay. **(A)** *V. parahaemolyticus* WT, Δ*VpdsbA1*, Δ*VpdsbA2*, and Δ*VpdsbA1/2* strains were tested in cadmium sensitivity assays by spotting 10^3^ cfu of each bacterial strain onto LB-NaCl containing increasing concentrations of cadmium (0–1.25 mM) in the presence or absence of 1 mM TC. **(B)** Motility assay of *V. parahaemolyticus dsb* mutants and *E. coli* Δ*EcdsbA* complemented with either EcdsbA, VpDsbA1, or VpDsbA2. **(C)** Quantification of the diameter of the motility circle of each strain shown in panel **(B)** was performed using ImageJ software. Graph represents percentages of each *V. parahaemolyticus dsb* mutant versus WT strain or *E. coli* Δ*EcdsbA* complemented with either VpDsbA1 or VpDsbA2 versus that with EcdsbA. Statistical analysis was calculated by one-way ANOVA. *n* = 3. ^∗∗^*P* < 0.001.

It was previously reported that *E. coli* Δ*dsbA* showed cadmium sensitivity due to the high affinity of Cd^2+^ for protein free thiols (Vallee and Ulmer, 1972). We tested *V. parahaemolyticus dsbA* mutant cadmium sensitivity as an indicator of oxidase capacity. *V. parahaemolyticus* WT and mutants were grown in the presence of increasing concentrations of cadmium (0–1.25 mM). The wild type strain of *V. parahaemolyticus* grew on concentrations of up to 1 mM cadmium, but the Δ*VpdsbA1*, Δ*VpdsbA2*, and Δ*VpdsbA1/2* mutants were cadmium-sensitive (Figure 3A and Table 3). These results suggest that both VpDsbA1 and VpDsbA2 possess oxidase activity.

**Table 3:** Cadmium sensitivity assay of *V. parahaemolyticus* WT and mutant strains.

	Mean cfu^b^ ± SD^c^		
Strains^a^	0.75^d^	1	1.25
**−TC**			
WT	46 ± 13	24 ± 5	0 ± 0
Δ*VpdsbA1*	47 ± 8	17 ± 8	0 ± 0
Δ*VpdsbA2*	56 ± 9	10 ± 2	0 ± 0
Δ*VpdsbA1/2*	2 ± 1	0 ± 0	0 ± 0
CΔ*VpdsbA1*	72 ± 7	34 ± 6	10 ± 2
CΔ*VpdsbA2*	74 ± 7	21 ± 4	0 ± 0
**+TC**			
WT	39 ± 4	26 ± 9	0 ± 0
Δ*VpdsbA1*	29 ± 4	0 ± 0	0 ± 0
Δ*VpdsbA2*	29 ± 1	0 ± 0	0 ± 0
Δ*VpdsbA1/2*	0 ± 0	0 ± 0	0 ± 0
CΔ*VpdsbA1*	29 ± 3	19 ± 4	0 ± 0
CΔ*VpdsbA2*	25 ± 3	3 ± 1	0 ± 0

*^a^The strains used were V. parahaemolyticus wild type (WT) and dsbA mutants, ΔVpdsbA1, ΔVpdsbA2, and ΔVpdsbA1/2, and the complementary strains CΔVpdsbA1 and CΔVpdsbA2 which contain P_BAD_-VpDsbA1 or P_BAD_-VpDsbA2, respectively. ^b^10^3^ cfu of each bacterial strain from the mid-log culture were spotted onto LB-NaCl containing 0.2% (wt/vol) of arabinose and increasing concentrations of cadmium in the presence (+TC) or absence (−TC) of 1 mM TC and incubated at 37°C for another 16 h. Colony-forming units (cfu) were quantified at each cadmium concentration. ^c^Experiments were performed in triplicate. ^d^Cadmium concentration (mM).*

*E. coli* Δ*dsbA* or Δ*dsbB* were reported to be defective in motility because they fail to assemble flagella due to a lack of disulfide bonds in the P-ring protein (FlgI) (Dailey and Berg, 1993). However, we did not observe the same motility defect in *V. parahaemolyticus dsb* mutants (Figure 3B,C). To test if VpDsbA1 and VpDsbA2 can rescue this defect in *E. coli* Δ*dsbA*, we introduced VpdsbA1 or VpdsbA2 on plasmids with a low copy number replication origin under the control of the P*_BAD_* promoter in *E. coli* Δ*dsbA*. Both VpDsbA1 and VpDsbA2 were able to restore the motility of *E. coli* Δ*dsbA* (Figure 3B,C).

### Bile Salts Repress VpDsbAs Activity

**FIGURE 4:**
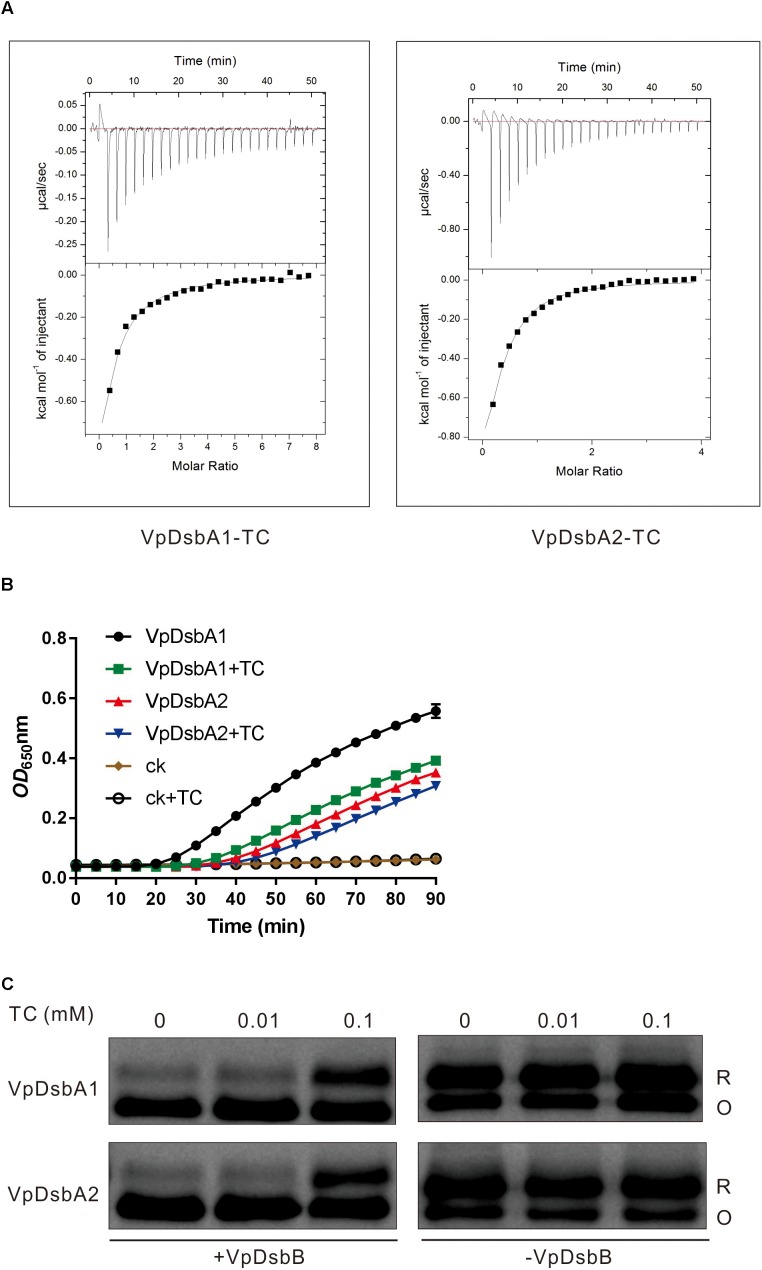
Bile salts inhibit VpDsbAs activity. **(A)** Bile salts interact with VpDsbA. ITC data titrating 4 mM of TC into 0.2 mM of tested proteins, VpDsbA1 (left) and VcDsbA2 (right). **(B)** Insulin reduction by VpDsbA1 or VpDsbA2 was inhibited in the presence of TC. Insulin (170 μM) and 0.33 mM DTT was incubated with 10 μM of VpDsbA, and 0.1 mM of TC were added at the beginning of reaction. The reduction of insulin was measured by monitoring the increase in absorbance at OD_650_. **(C)** Reduced VpDsbA oxidized by VpDsbB present in the membranes *in vitro*. Reduced VpDsbA (2 μM) was incubated with or without VpDsbB containing membranes and trapped with AMS after various incubation times. After incubation with VpDsbB membrane, VpDsbA (O) shifts to a lower molecular weight band. VpDsbA was detected using Western blot with an anti-VpDsbA antibody.

When *V. parahaemolyticus* enters into the human body through the consumption of contaminated water or uncooked food, a set of virulence determinants controlled by a regulatory network are produced in response to the chemical signals present in the small intestine where it normally causes disease (Rivera-Cancel and Orth, 2017). *V. parahaemolyticus* uses bile salts to regulate virulence factor expression (Gotoh et al., 2010; Li et al., 2016). Bile salts also induce virulence gene expression in *V. cholerae* by interfering with the redox reaction of DsbA proteins (Xue et al., 2016). We first investigated the interaction between the bile salt TC and VpDsbA1 and VpDsbA2 by using ITC. We found that both VpDsbA1 and VpDsbA2 can bind TC, with a *K_D_* of 131 ± 7 μM and 164 ± 11 μM, respectively (Figure 4A). We also tested TrxA from *E. coli* binding to TC by ITC, but EcTrxA and TC did not show any specific interaction (Supplementary Figure S2) which indicated that the binding of VpDsbA1 or VpDsbA2 with TC is a specific interaction event.

Insulin reduction mediated by either VpDsbA1 or VpDsbA2 was inhibited by TC (Figure 4B). The cadmium sensitivity assay showed that *V. parahaemolyticus* was more sensitive to cadmium in the presence, than in the absence of TC (Figure 3A and Table 3), indicating that TC might reduce the efficiency of disulfide bond formation catalyzed by VpDsbAs in the periplasm of *V. parahaemolyticus*. DsbA reductase activity correlates with DsbA reoxidation by DsbB (Ren et al., 2009). To test if VpDsbA activity inhibited by TC *in vivo* is because TC interferes with the reaction between VpDsbA and VpDsbB, we studied the oxidization of VpDsbA by VpDsbB *in vitro*. The oxidization of both VpDsbA1 and VpDsbA2 by the membrane protein VpDsbB was inhibited by TC *in vitro* (Figure 4C).

### VpDsbAs Impact *V. parahaemolyticus* Pathogenesis

**FIGURE 5:**
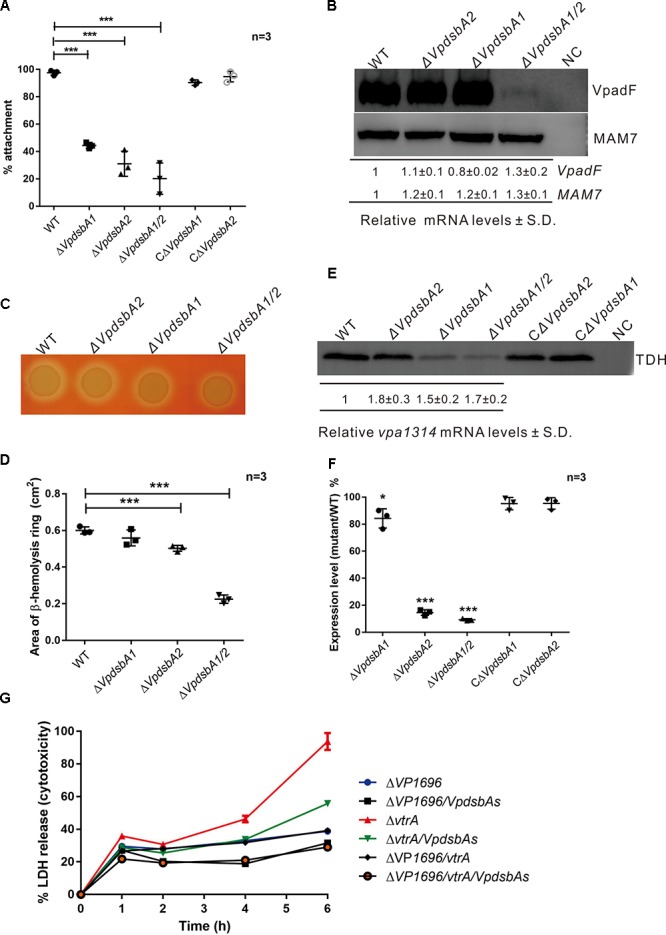
VpDsbAs is required in pathogenesis of *V. parahaemolyticus*. **(A)** Adhesion of *V. parahaemolyticus* WT, Δ*VpdsbA1*, Δ*VpdsbA2*, and Δ*VpdsbA1/2* and the complement strains to Caco-2 cells. Statistical analysis was calculated by one-way ANOVA. *n* = 3. ^∗∗∗^*P* < 0.001. **(B)** *Top*, analysis of VpadF and MAM7 protein levels. *V. parahaemolyticus* WT, Δ*VpdsbA1*, Δ*VpdsbA2*, and Δ*VpdsbA1/2* containing P*_BAD_*-VpadF-cFLAG or P*_BAD_*-MAM7-cFLAG were grown in LB-NaCl until OD_600_≈0.8. Cell lysates (1 mg) were separated by SDS-PAGE and VpadF or MAM7 was detected by the Western blot using anti-FLAG monoclonal antibody (Sigma-Aldrich, United States). Blot shown is representative of at least three separate experiments. *Bottom*, analysis of *VpadF* and *MAM7* mRNA levels by qRT-PCR. RNA was purified from freshly prepared cultures grown in LB-NaCl. The relative mRNA levels ± SD were normalized to 16S RNA. **(C)** Hemolysis activity of *V. parahaemolyticus* WT, Δ*VpdsbA1*, Δ*VpdsbA2*, and Δ*VpdsbA1/2* strains on Wagatsuma blood agar. **(D)** Quantification of area of the hemolysis circle of each strain shown in panel **(B)** was performed using ImageJ software. Statistical analysis was calculated by one-way ANOVA. *n* = 3. ^∗^*^∗∗^P* < 0.001. **(E)** *Top*, analysis of TDH protein level. *V. parahaemolyticus* WT, Δ*VpdsbA1*, Δ*VpdsbA2*, and Δ*VpdsbA1/2* and the complement strains were kept growing in LB-NaCl containing 1 mM of taurodeoxycholate acid (TDCA) at 37°C until 10% (vol/vol) trichloroacetic acid (TCA) was added. Protein samples were suspended in a buffered solution containing 100 mM Tris-HCl pH 7.5 and 1% (wt/vol) SDS and 1 mg of each sample was separated by SDS-PAGE and TDH was detected by the Western blot using anti-TDH antibody. Blot shown is representative of at least three separate experiments. *Bottom*, analysis of *vpa1314* mRNA levels by qRT-PCR. RNA was purified from freshly prepared cultures grown in LB-NaCl containing 1 mM of TDCA. The relative mRNA levels ± SD were normalized to 16S RNA compared with that of WT. **(F)** Quantification of band intensities from blot shown in panel **(E)** was performed using ImageJ software. Graph represents percentages of TDH compared with that of WT. Protein expression levels were normalized to that of WT. Data shown are averages of three independent experiments. Statistical analysis was calculated by one-way ANOVA. *n* = 3. ^∗^*P* < 0.05, ^∗∗∗^*P* < 0.001. **(G)** Cytotoxicity assay of *V. parahaemolyticus* mutant strains against HeLa cells by detecting the release of LDH into the medium at each indicated time. Parameters reported include the mean ± SD across three replicates.

*Vibrio parahaemolyticus* has evolved several regulatory networks that control the production of a wide range of virulence factors which enables them to cause disease in the host, including the thermostable direct hemolysin (tdh) and TDH related hemolysin (trh), adhesin, and secreted effectors (Zhang and Orth, 2013; Liu and Chen, 2015). We found that *V. parahaemolyticus*Δ*VpdsbA1/2* exhibits reduced virulence in the zebrafish infection model (Figure 1C). To understand why, we first investigated the effect of VpDsbAs on host cell adhesion (Liu and Chen, 2015). Deleting either *VpdsbA1* or *VpdsbA2* decreased *V. parahaemolyticus* attachment to Caco-2 epithelial cells (Figure 5A). Several adhesion factors present at the surface of *V. parahaemolyticus* have been reported to be important in host cell binding, including MAM7 (Multivalent Adhesion Molecule 7) (Krachler et al., 2011), MSHA pilus (O’Boyle et al., 2013), PilA (Shime-Hattori et al., 2006), and VpadF (Liu and Chen, 2015). To determine whether VpDsbAs are involved in maintaining the stability of these adhesion factors, we quantified the abundance of MAM7 (VP1611), MshA (VP2697), PilA (VP2523), and VpadF (VP1767). The abundance of VpadF, but not MAM7 was significantly reduced in the Δ*VpdsbA1/2* mutant but not in Δ*VpdsbA1* or Δ*VpdsbA2* single knock-out mutant (Figure 5B) which indicates that either one of these two VpDsbA proteins is necessary for VpadF stability. The VpDsbAs had no effect on the transcription levels of all these adhesion factors (Figure 5B and Supplementary Figure S3). These results indicate that the stability of VpadF depends on a functional DsbA protein present in the periplasm of *V. parahaemolyticus* which plays an important role in *V. parahaemolyticus* adhesion to epithelia cells.

*Vibrio parahaemolyticus* strains isolated from clinical samples are able to lyse human erythrocytes when plated on a high-salt media called Wagatsuma agar, a process termed the Kanagawa (KP) test (Nishibuchi and Kaper, 1995). To determine whether VpDsbAs contribute to the β-hemolytic activity of *V. parahaemolyticus*, we performed the KP test with WT and the *dsbA* mutants. We found that the β-hemolytic activity significantly decreased when both *VpdsbA* genes are deleted (Figure 5C,D). TDH is the major toxin that contributes to the β-hemolytic activity of *V. parahaemolyticus* (Honda et al., 1988; Nishibuchi et al., 1992). The mRNA level of TDH was not affected by VpDsbAs (Figure 5E,F). However, the amount of TDH protein decreased significantly in the Δ*VpdsbA1* and Δ*VpdsbA1/2* mutants (Figure 5E,F), suggesting that VpDsbA1 plays a major role in maintaining TDH stability.

*Vibrio parahaemolyticus* harbors two type III secretion systems (T3SS) encoded in chromosomes 1 (T3SS1) and 2 (T3SS2) (Makino et al., 2003). T3SS1 is responsible for cytotoxicity and T3SS2 is primarily involved in enterotoxicity, as well as in cytotoxic activity against some specific cell lines (Hiyoshi et al., 2010). To test whether VpDsbAs affect cytotoxicity of *V. parahaemolyticus* against HeLa cells, HeLa cell lysis was measured by monitoring the release of LDH after infection with *V. parahaemolyticus tdh*As and T3SS deletion mutant strains in the presence or absence of VpDsbAs. VtrA is the master regulator of T3SS2 in *V. parahaemolyticus*, so T3SS2 will stop working with *vtrA* deletion (Kodama et al., 2010); and T3SS1 will lose function when the inner membrane protein VP1696 is deleted from the genome (Park et al., 2004). Consistent with previous reports (Park et al., 2004; Hiyoshi et al., 2010), T3SS1 of *V. parahaemolyticus* works dominantly in cytotoxicity of HeLa cells (Figure 5G). After 6 h infection, HeLa cells were nearly completely lysed by *V. parahaemolyticus* T3SS2 mutant strains containing a functional T3SS1, but strains without VpDsbAs showed ∼40% less lysis (Figure 5G). This suggests that VpDsbAs play an important role in epithelial cell infection through T3SS1. By assessing the transcription level and the secretion level of some major effectors and the activators of T3SS1, we found that VpDsbAs do not have a significant effect on the transcription of genes involved in T3SS1 activity (Supplementary Figure S4). However, the secretion efficiency of VPA0450 decreased in *dsbA* mutant compared with that in WT strain, while VP1683 was secreted more efficiently in the mutant strain under the conditions that we tested (Supplementary Figure S5). We did not figure out what’s the mechanism that VpDsbAs work differently in the secretion of these two effectors, yet, this strongly suggests that VpDsbAs affect T3SS1 functions at the post-translational level.

## Discussion

DsbA participates in protein folding by introducing disulfide bonds into proteins secreted to the periplasm (Hu et al., 1997; Heras et al., 2009). In this study, we characterized the redox properties of two DsbA proteins from *V. parahaemolyticus* and investigated their essential role for several important virulence factors that affect *V. parahaemolyticus* pathogenesis.

Two DsbA genes, VpDsbA1 and VpDsbA2, are encoded on *V. parahaemolyticus* chromosomes 1 and 2, respectively. Both genes share high sequence similarity with that of VcDsbA and EcDsbA and contain the classical CXXC active-site motif and *cis*Pro motif (Figure 1A), catalyzed insulin reduction *in vitro* (Figure 2A), and introduced disulfide bonds to the proteins secreted to the periplasm of *V. parahaemolyticus in vivo* (Figure 3A). Unlike *E. coli* or *V. cholerae*, the motility of which is mainly driven by one set of flagellar system, *V. parahaemolyticus* possesses dual flagellar systems, a single polar flagellum which propels the bacterium in liquid (swimming) and the lateral flagella which drive the bacterium move on the surface (swarming) (McCarter and Silverman, 1990; McCarter, 2004). *V. parahaemolyticus dsb* mutant strains were still motile (Figure 3B). We speculate that if the polar flagellum stops working, which might be the case in the *dsb* mutants, the lateral flagella will be activated and enable *V. parahaemolyticus* to remain motile (McCarter et al., 1988; McCarter and Silverman, 1989).

Like *V. cholerae* which activates virulence production by sensing bile salts (Yang et al., 2013; Xue et al., 2016), *V. parahaemolyticus* also hijacks bile salts as an intestinal signal to regulate virulence production (Gotoh et al., 2010). *V. parahaemolyticus* activates T3SS2 gene expression by sensing bile salts taurodeoxycholate acid (TDCA; Rivera-Cancel and Orth, 2017). Here, we found that both VpDsbA1 and VpDsbA2 can bind TC and TC repressed disulfide bond formation in the periplasm of *V. parahaemolyticus* by inhibiting the reoxidation of VpDsbA1 or VpDsbA2 by VpDsbB (Figure 4). Bile salts act as a stressor to bacteria that transit the intestinal tract, so it is reasonable to speculate that bile salts might regulate *V. parahaemolyticus* bile resistance by affecting the activity of the Dsb system.

This study also describes the essential role of the two DsbA proteins for several important virulence factors that affect *V. parahaemolyticus* pathogenesis. Both VpDsbA1 and VpDsbA2 are transcribed and expressed in *V. parahaemolyticus* under the conditions that we tested. *V. parahaemolyticus dsbA* mutants, especially the double mutant, showed defects in attachment to Caco-2 cells, β-hemolytic activity, and cytotoxicity against HeLa cells (Figure 5). All the mutants of Δ*VpdsbA1*,Δ*VpdsbA2*, and Δ*VpdsbA1/2* showed defects in attachment to Caco-2 cells compared with that of the wild type strain (Figure 5A), however, the amount of adhesion factor VpadF decreased dramatically only in Δ*VpdsbA1/2* mutant and either VpDsbA1 or VpDsbA2 protein alone was sufficient for VpadF stability (Figure 5B). All the other adhesion factors that we tested in this study are not affected by VpDsbA1 or VpDsbA2. This might indicate that some other unknown adhesion factors which are required VpDsbAs to fold correctly also contribute to the adhesion of *V. parahaemolyticus* to mammal cells. Compared to VpDsbA2, VpDsbA1 played a more important role in affecting the stability of virulence factor TDH (Figure 5D) in *V. parahaemolyticus*. However, TDH can be oxidized by either VpDsbA1 or VpDsbA2 *in vitro* (Supplementary Figure S6). So VpDsbA1 and VpDsbA2 might work differently in maintaining TDH protein stability *in vivo*.

*Vibrio parahaemolyticus dsbA* mutant also showed dramatically reduced cytotoxicity against HeLa cells in the T3SS2 knock-out background (containing functional T3SS1) (Figure 5E). Although the molecular mechanism by which VpDsbAs affect T3SS1 is not clear yet, it is reasonable to assume that the catalytic activity of VpDsbA to form a disulfide bond for the functional folding of various proteins is required for its virulence. *V. parahaemolyticus* T3SS1 is similar to the Ysc secretion system in *Yersinia* (Troisfontaines and Cornelis, 2005). *Yersinia pestis* DsbA is required for the formation of a ring-shaped structure in a type III secretion apparatus to secrete virulence effectors (Jackson and Plano, 1999). Like *Yersinia*, the DsbA proteins of *V. parahaemolyticus* might also be required for the formation of the type III secretion apparatus. VcDsbA from *V. cholerae* was reported to be indispensible for the pathogenesis of this organism. VcDsbA is required for the functional maturation of secreted virulence factors in *V. cholerae* (Peek and Taylor, 1992; Hu et al., 1997). VcDsbA is also essential for the homodimerization of TcpP to activate virulence gene expression in *V. cholerae* (Yang et al., 2013; Xue et al., 2016). VtrA, which is the master regulator of T3SS2 genes in *V. parahaemolyticus*, adopts the same topology and function as TcpP (Rivera-Cancel and Orth, 2017). The *dsbA* mutants did not show a defect in the cell invasion assay; on the contrary, the efficiency of invasion to HeLa cells was increased without VpDsbA (Supplementary Figure S7). We have checked the mRNA level of some T3SS2 relevant genes, *vpa1321*, *vpa1327*, *vpa1332*, *vpa1346*, *vpa1348* and *vpa1370* all of which have been reported to be important for T3SS2 function (Broberg et al., 2011; Kodama et al., 2015). We found that in the presence of TDCA, the mRNA level of all these tested genes in *dsbA* mutant did not show much difference from that of WT (Supplementary Figure S8). All these indicate that VpDsbAs might somehow affect T3SS2 function through an unknown mechanism.

Overall, this study describes the roles of DsbA in host cell adhesion, cytotoxicity, and β-hemolytic activity, which suggests that VpDsbAs could be a potential target for the development of antibacterial compounds to control *V. parahaemolyticus* infections.

## Data Availability

This manuscript contains previously unpublished data. The name of the repository and accession number are not available.

## Ethics Statement

All procedures performed in studies involving animals were in accordance with the ethical standards of the Institutional Animal Care and Use Committee of Zhejiang A&F University at which the studies were conducted (Permit Number: ZJAFU/IACUC_2011-10-25-02).

## Author Contributions

C-qW, TZ, WZ, MS, FT, AY, and ML performed the research (the acquisition, analysis, or interpretation of the data). C-qW, TZ, WZ, and MY analyzed the data. MY designed the research and wrote the manuscript.

## Conflict of Interest Statement

The authors declare that the research was conducted in the absence of any commercial or financial relationships that could be construed as a potential conflict of interest.

## Footnotes

1 http://zfin.org/zf_info/zfbook/zfbk.html

2 http://rsbweb.nih.gov/
