# Supplementary material for: Two DsbA Proteins Are Important for Vibrio parahaemolyticus Pathogenesis
Source: Front Microbiol. 2019 May 16;10:1103. doi: 10.3389/fmicb.2019.01103 (PMC6531988; doi:10.3389/fmicb.2019.01103)
Supplement: Supplementary file 1 [file Data_Sheet_1.docx]

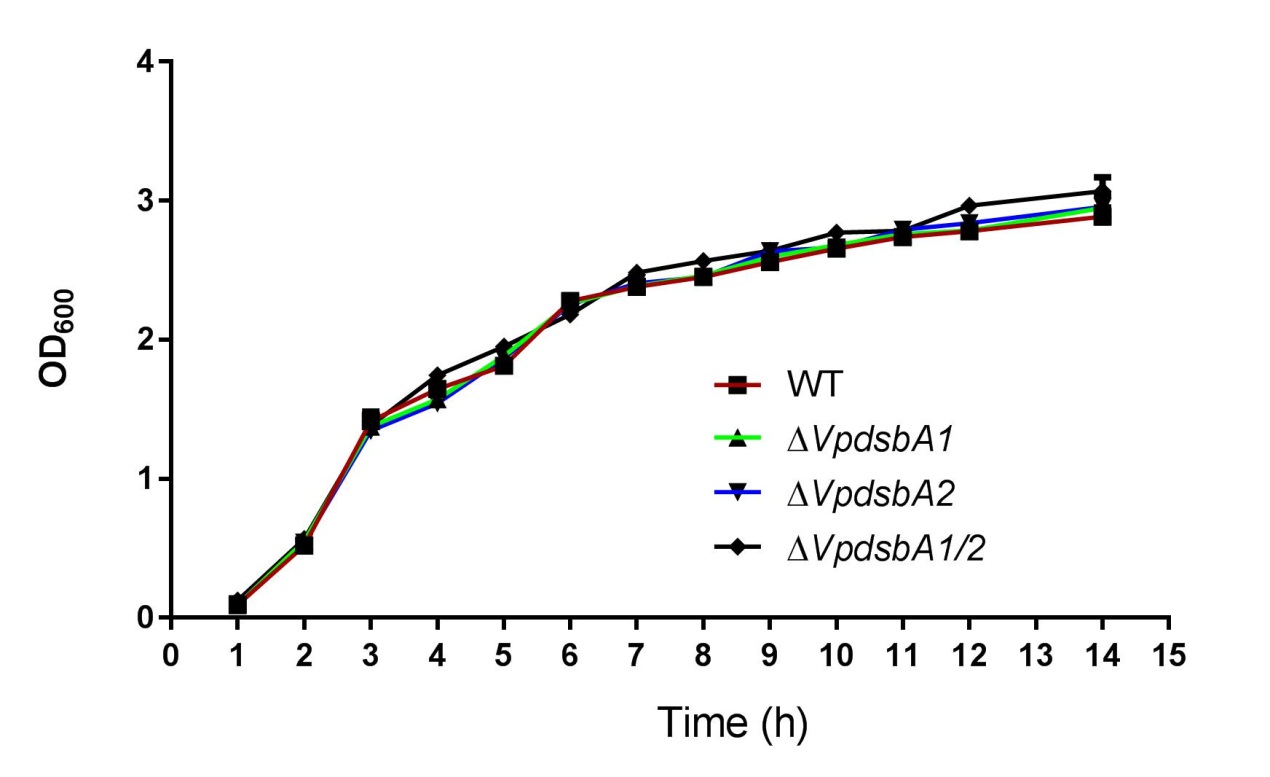


Fig. S1. Growth assay of *V. parahaemolyticus* WT, Δ*VpdsbA1*, Δ*VpdsbA2* and Δ*VpdsbA1/2* strains.


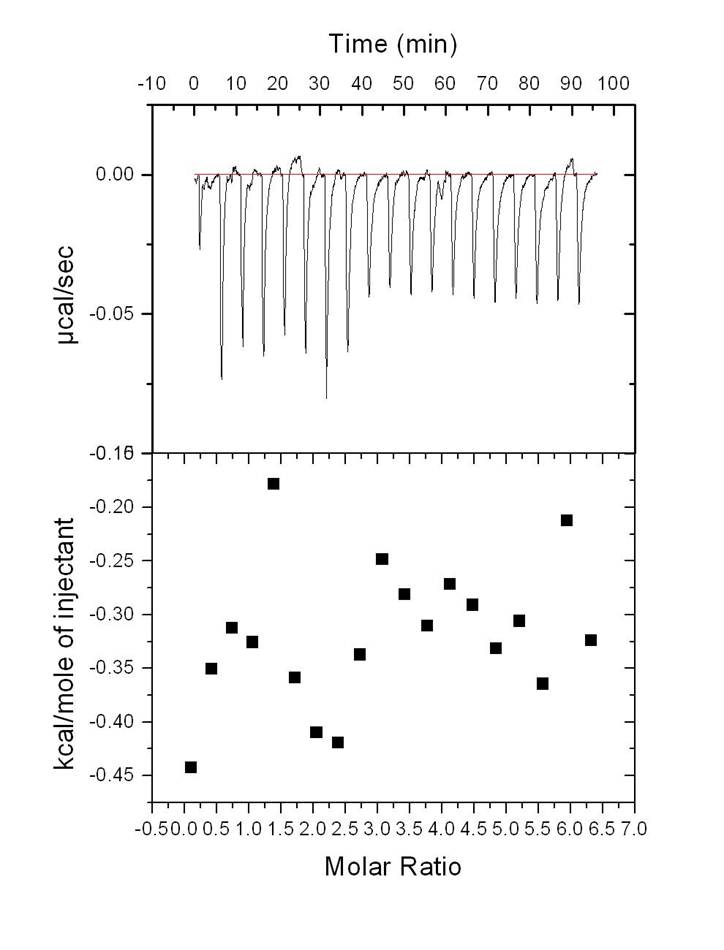


Fig. S2. ITC data titrating 4 mM of TC into 0.2 mM of EcTrxA.


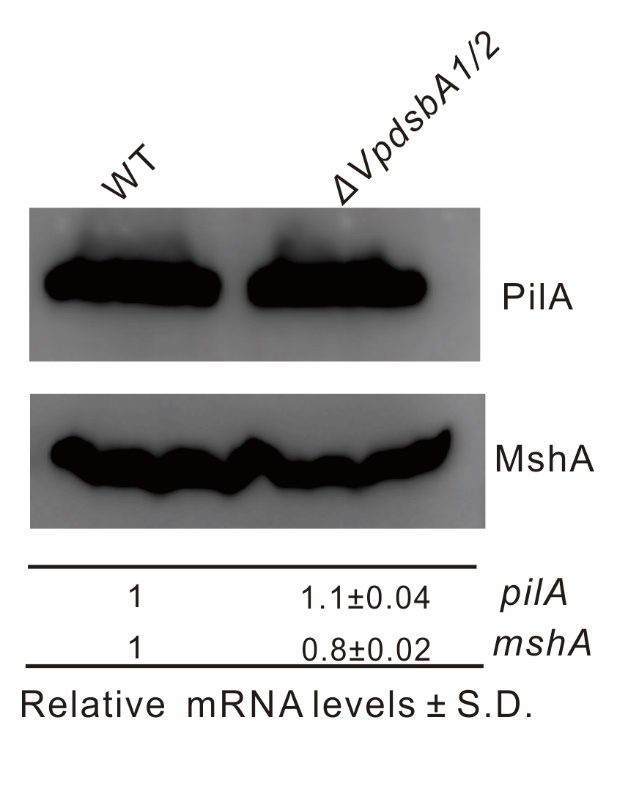


Fig. S3. Expression level assay of *V. parahaemolyticus* PilA and MshA. *Top*, analysis of VpadF and MAM7 protein level. *V. parahaemolyticus* WT, Δ*VpdsbA1*, Δ*VpdsbA2* and Δ*VpdsbA1/2* containing P*_BAD_*-PilA-cFLAG or P*_BAD_*-MshA-cFLAG were grown in LB-NaCl until OD_600_≈0.8. Cell lysates (1 mg) were separated by SDS-PAGE and VpadF or MAM7 was detected using Western blotting with an anti-FLAG monoclonal antibody (Sigma). Blot shown is representative of at least three separate experiments. *Bottom*, analysis of *pilA* and *mshA* mRNA levels by qRT-PCR. RNA was purified from freshly prepared cultures grown in LB-NaCl. The relative mRNA levels ± S.D. were normalized to 16sRNA.


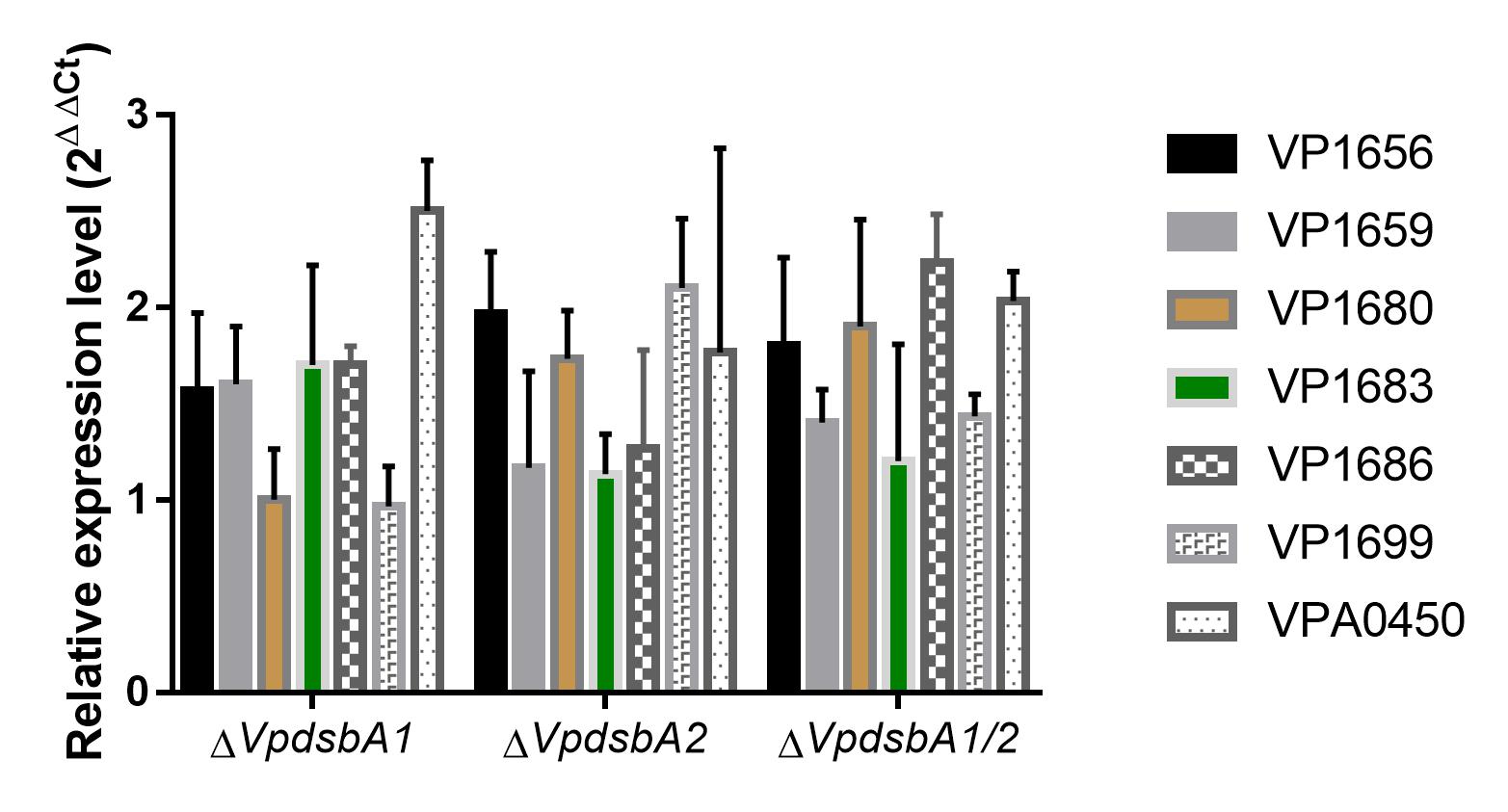


Fig. S4. mRNA level assay of *V. parahaemolyticus* T3SS relevant genes by RT-PCR. RNA of *V. parahaemolyticus* WT, Δ*VpdsbA1*, Δ*VpdsbA2* and Δ*VpdsbA1/2* strains was purified from freshly prepared cultures grown in LB-NaCl. The relative mRNA levels ± S.D. were normalized to 16s RNA compared with that of WT.


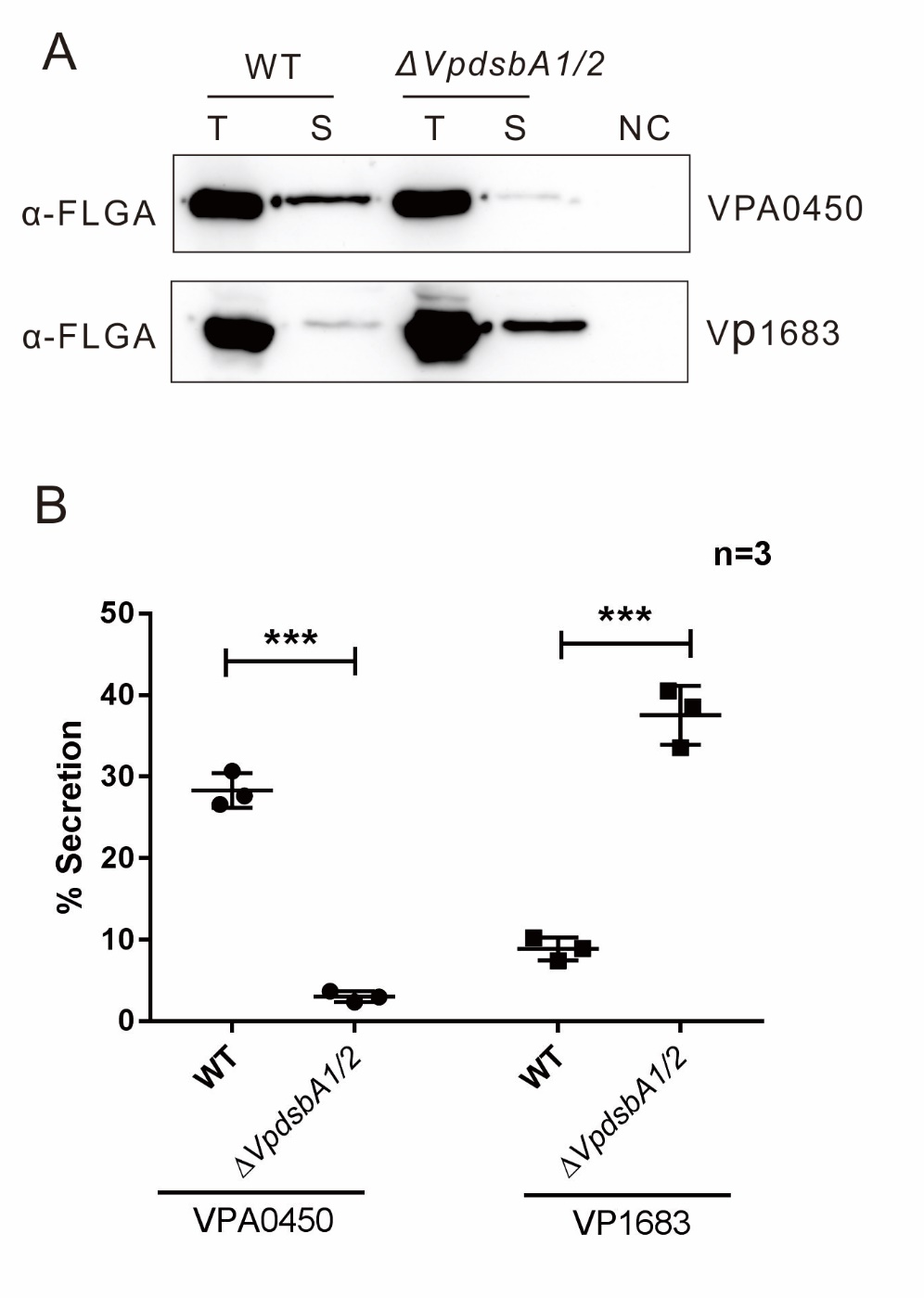


Fig. S5. Expression level assay of *V. parahaemolyticus* VPA0450 and VP1683. A. *V. parahaemolyticus* WT and Δ*VpdsbA1/2* containing P*_BAD_*-VPA0450-cFLAG or P*_BAD_*-VP1683-cFLAG were grown in LB-NaCl with 0.2% (w/v) of arabinose at 37 ˚C for around 12 h until OD_600_≈2.0. 10^8^ cells from each bacterial culture (T) or supernatant (S) were precipitated by 10% (w/v) TCA at 4 ˚C overnight and then separated by SDS-PAGE and VPA0450 and VP1683 was detected using Western blotting with an anti-FLAG monoclonal antibody (Sigma). Blot shown is representative of at least three separate experiments. B. Quantification of band intensities from blot shown in panel A was performed using ImageJ software. Graph represents percentages of the secretion protein (S) compared with the whole cell protein from the total bacterial culture (T). Data shown are averages of three independent experiments. Statistical analysis was calculated by 1-way ANOVA. n=3. *** *P* < 0.001.


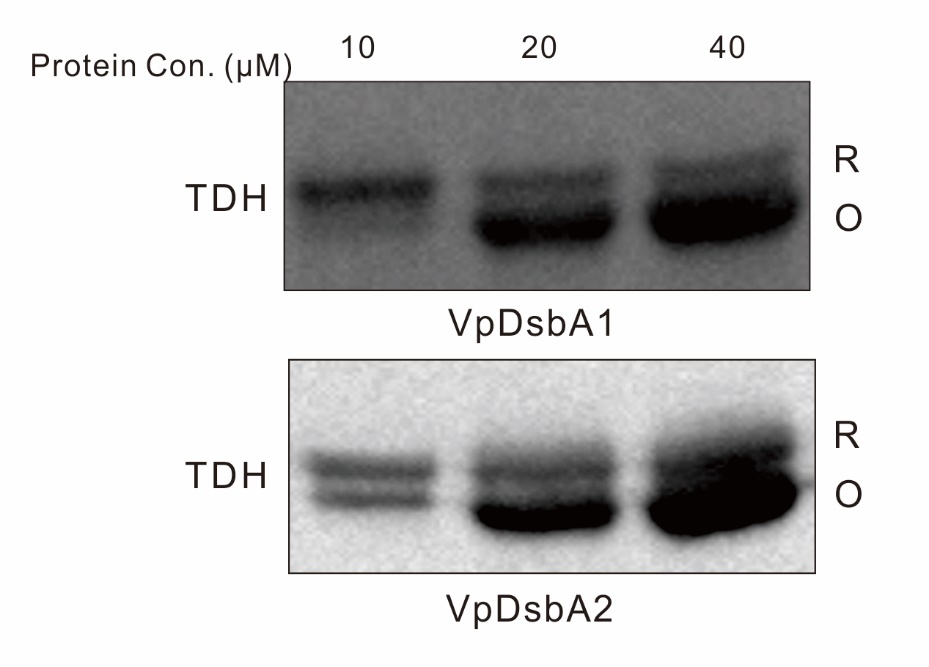


Fig. S6. Oxidation of reduced TDH by oxidized VpDsbA1 or VpDsbA2 in vitro. Reduced TDH (2 μM) was incubated with oxidized VpDsbA1 or VpDsbA2 and trapped with AMS after incubating at room temperature for 30 min. Oxidized TDH (O) shifts to a lower molecular weight band. TDH was detected by Western blot using anti-TDH antibody.


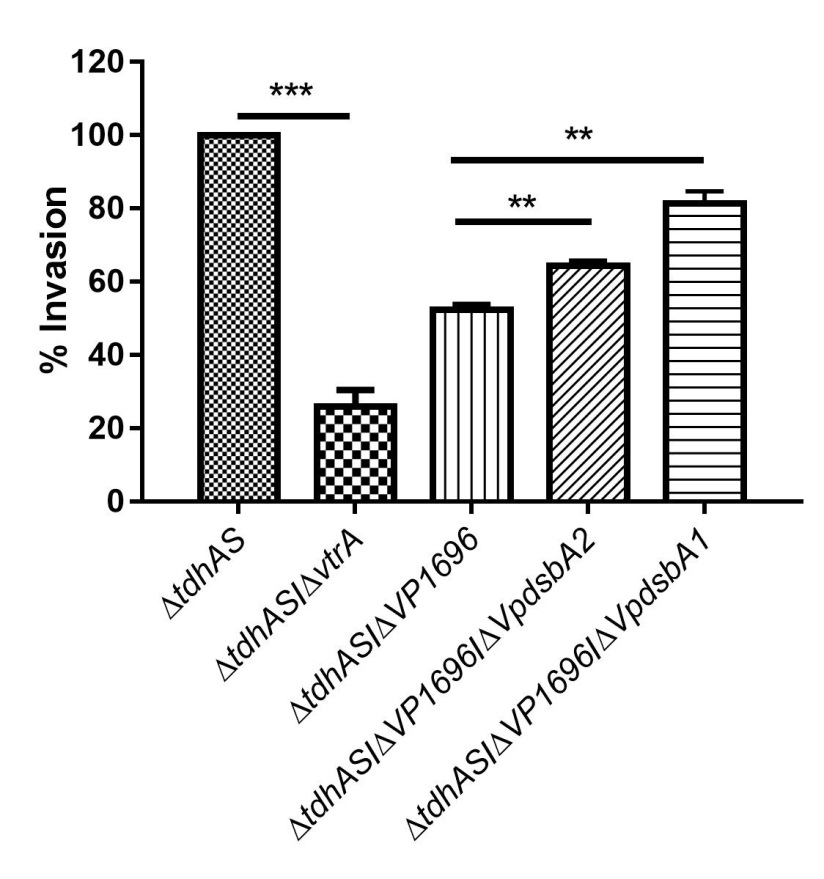


Fig. S7. Invasion assay of *V. parahaemolyticus* mutant strains in Hela cells. Over-night cultured bacteria were sub-cultured at 1:100 into the fresh LB-NaCl medium until the OD_600_ reached 0.6. Bacterial cells were added to the HeLa cell monolayers at a MOI of 10 and incubated for 120 min at 37 ˚C and 5 % CO_2_. After washing three times with PBS, 1mL DMEM medium containing 100 μg/ml gentamicin was added to the infected cells and incubated for an additional 60 min to kill extracellular bacteria. HeLa cells were washed with PBS for three times, and then lysed with 500 μl 0.1% Triton X-100. Bacteria release from HeLa cell were then enumerated by CFU counting on LB-NaCl agar plates. Invasion were calculated as percentages of the number of bacteria recovered from the total bacteria inoculated. All assays were performed with at least three independent biological replicates. ** *P* < 0.01, *** *P* < 0.001 (Student t test).


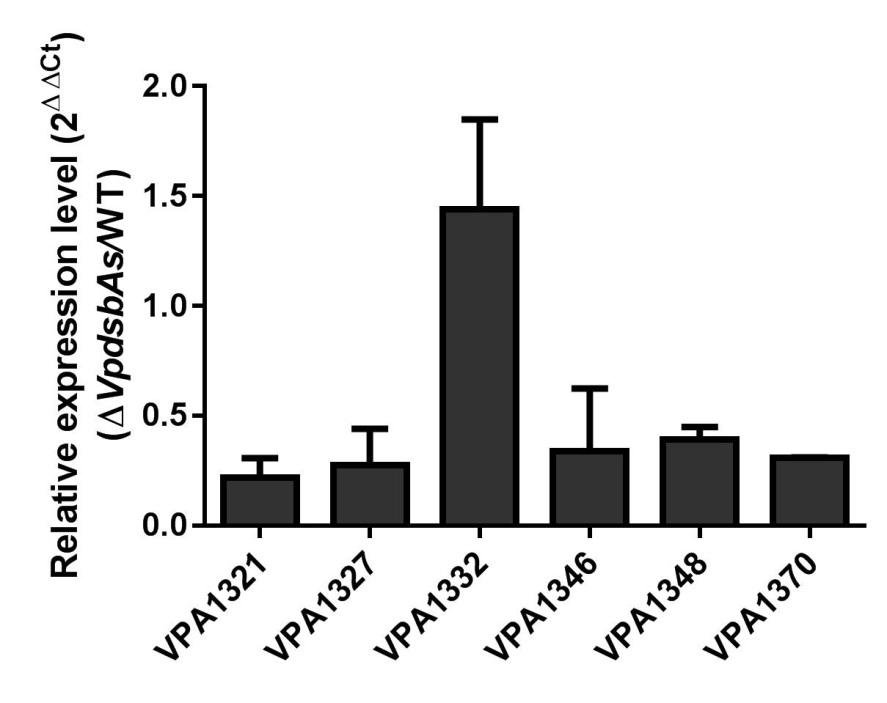


Fig. S8. mRNA level assay of *V. parahaemolyticus* T3SS2 relevant genes by RT-PCR. RNA of *V. parahaemolyticus* WT and Δ*VpdsbA1/2* strains was purified from freshly prepared cultures grown in LB-NaCl containing 1 mM of taurodeoxycholate acid. The relative mRNA levels ± S.D. were normalized to 16s RNA compared with that of WT.
